# Public Health Chemistry and Bacteriology

**Published:** 1913-03

**Authors:** 


**Public Health Chemistry and Bacteriology.** By David McKail, M.D., D.P.H Pp. vii, 409. Bristol: John Wright & Sons Limited. 1912. 6s. 6d. net.

Dr. McKail describes this book as a handbook for D.P.H. students, and explains in his preface that it is based on notes prepared by the writer for his classes. Standard works on the subjects with which it deals and also papers in current journals have been freely used, and the amount of matter contained in a comparatively small compass is certainly considerable. The book is divided into two parts, chemical and bacteriological, and there are appendices dealing with the regulations for the D.P.H., with the Local Government Board Regulations as to the use of preservatives in milk and cream, and with Park and Krumwiede's studies on bovine and human types of tubercle bacilli. There is also an efficient index. The descriptions are necessarily brief, but quite lucid and complete, and will be readily followed by those who have experience of laboratory methods The book is in no sense a " cram book," and could not, we think, under any circumstances be used as such ; it may be confidently recommended to those who are studying Public Health in the usual laboratory courses as a useful adjunct to class work, and as a work of reference. The theoretical matter is very briefly dealt with, but is sufficiently complete to prove useful in revision work, or as complementary to instruction already received in classes or lectures, but would be found somewhat too concise for those approaching the subject for the first time. The practical methods are described in sufficient detail to be useful as laboratory guides, and are quite up to date. As a whole we consider the book a good one, as it covers a large field and will be found quite reliable in detail.

